# Measurement of compassion fatigue in animal health care professionals: a systematic review of available instruments and their content validity

**DOI:** 10.3389/fvets.2024.1425741

**Published:** 2024-07-26

**Authors:** May Thet Nu Noe, Yigit Baysal, Anaïs Masserey, Sonja Hartnack, Irina Guseva Canu

**Affiliations:** ^1^Centre for Primary Care and Public Health (Unisanté), University of Lausanne, Lausanne, Switzerland; ^2^Section of Epidemiology, Vetsuisse Faculty, University of Zurich, Zurich, Switzerland

**Keywords:** compassion fatigue, patient-reported outcome measures, content validity, mental health, animal health care professionals

## Abstract

**Introduction:**

Compassion fatigue (CF) refers to emotional or physical exhaustion and emotional reactions resulting from prolonged exposure to traumatic events, commonly experienced by professionals in caregiving roles. CF is prevalent among healthcare professionals, including those in animal care. Several Patient-Reported Outcome Measures (PROMs) were developed to measure CF, but their psychometric validity was not reviewed systematically. This study aims to identify and review the content validity of CF PROMs used in animal health care professionals.

**Methods:**

Literature was searched in PubMed, PsycINFO, and EMBASE (1973–2023). We included studies conducted in animal health care professionals, using a PROM to measure CF, reporting at least one psychometric property of this PROM, and published as original research. For each identified PROM, additional literature search was conducted to identify PROM development and content validation studies. Three independent reviewers evaluated the content validity of each PROM using COnsensus-based Standards for the selection of health Measurement INstruments (COSMIN) methodology and summarized the quality of evidence using a modified GRADE approach. The protocol was registered in PROSPERO (CRD42023433982) and results reported following PRISMA guidelines.

**Results:**

Initially, 1709 studies were identified. After a double screening, 17 eligible studies were included. CF was measured using six different PROMs or their modified versions. Only one PROM specifically targeted animal health care professionals: the ProQOL-5 Veterinary Medicine Version. This and three other original CF PROMs were reviewed. For all PROMs, the quality of content validity was rated as insufficient due to deficiencies in the concept and items elicitation, inadequate target population representation, and inadequate details on cognitive interview procedures. The overall evidence quality was rated as low due to a limited number of PROM validation studies, poor methodological and reporting quality, and indirect result.

**Discussion:**

There is a scarcity of studies examining CF within the target population, and the quality of evidence for content validity of the reviewed PROMs for CF measurement is currently low. CF definition and construct description in PROM development studies suffer from vagueness and seem inadequately reflected by the content of the reviewed PROMs. Further research with a robust methodology seems necessary to address the identified flows.

**Systematic review registration:**

Measurement of compassion fatigue in people working with animals: protocol for a systematic review. PROSPERO 2023 CRD42023433982. Available from: https://www.crd.york.ac.uk/prospero/display_record.php?ID=CRD42023433982.

## 1 Introduction

Carla Joinson described the term Compassion Fatigue (CF) in 1992 and applied it to nurses. Joinson noted that behavioral signs of CF included chronic exhaustion, impatience, and a lack of joy in life (1). Figley introduced the term “secondary traumatic stress” (STS) to denote CF and characterized it as the “cost of caring” and “the emotional pain and exhaustion that occurs with a gradual onset that arises in some care providers when exposed to a suffering individual” (2). More recently, CF has been conceptualized as a multi-component construct including both burnout and secondary traumatic stress (3), bringing together various terms used in the field. CF and moral distress are examples of occupational mental health concerns that have been investigated in healthcare workers, but seldom in other professions with similar challenges, such as animal health care professionals (4–6).

Veterinary professionals and lab technicians that work with animals are particularly vulnerable to mental illness and suicide because of certain pressures such as euthanasia (4, 5, 7–11). Platt’s systematic review, titled “Suicidal behavior and psychosocial problems in veterinary surgeons” has uncovered numerous elements contributing to workplace stress within the veterinary field. These include extended working hours, deadline pressures, challenging interactions with clients, financial discussions with clients, delivering distressing news, experiencing moral and ethical dilemmas, maintaining an unbalanced work-life schedule, perfectionism, exposure to instances of animal abuse and euthanasia, and the transition from university to clinical practice (12). Euthanasia stress is a specific type of occupational stressor in animal care practice. Professionals directly involved in euthanasia exhibit elevated levels of work stress, diminished job satisfaction, increased turnover, and psychological distress (10). This phenomenon, known as the “caring-killing paradox,” highlights the unique stressors faced by these professionals due to the moral and emotional challenges of euthanizing animals they care for (13). Studies have indicated a correlation between the number of euthanized dogs and employee turnover, potentially associated with a decline in job satisfaction (14). Finally, euthanasia has been identified as an important component of CF (15). CF affects animal welfare and the 3R principle (replacement, reduction, refinement) in research (16), with limited control over euthanasia linked to lower quality of life in lab workers (17). Therefore, exploring CF in animal care workers is important.

This systematic review had two research objectives. The first objective was to identify the measures used for CF and its extent among animal health care professionals. The second objective was to assess the content validity of these measures, which is the most important psychometric property in the patient reported outcome measures (PROMs) (18). Indeed, CF being a subjectively measured outcome, in most cases it is addressed using a PROM, i.e., a standardized questionnaire designed to gather information directly from patients regarding health outcomes, encompassing aspects such as symptoms, health-related quality of life, and functional status (19). This two-fold approach aimed to not only catalog the CF PROMs employed in the field but also delve into their psychometric validity.

## 2 Materials and methods

### 2.1 Protocol and registration

The protocol of this systematic review was registered in the International Prospective Register of Systematic Reviews (PROSPERO) under the registration number CRD42023433982. This study was conducted following the CRD’s guidance for undertaking systematic reviews in the health care (20).The results were reported according the Preferred Reporting Items for Systematic Reviews and Meta-Analyses (PRISMA) (21).

### 2.2 Eligibility criteria

We included studies 1-conducted in animal health care professionals, 2-measuring the CF using a PROM, 3-assessing, and reporting at least one psychometric property of this PROM in the study sample, 4-published as original research articles in a peer-reviewed journal in English, German, French, Turkish, or Russian languages. All other studies and publication types were excluded.

#### 2.2.1 Study screening and selection

A systematic literature search was performed for the period 1973–2023 in three databases: PubMed, PsycINFO, and EMBASE. The research strategy consisted of free-text words to specify two search strings. The first string focused on terms related to animal-related occupations and CF, with a specific emphasis on PROM as a measurement tool. The second search strings terms are linked to psychometric properties. Both searched strings were developed by an experienced librarian in collaboration with two most experienced reviewers [SH, IGC].

The librarian imported the collected studies in the bibliography software EndNote 20 and removed the duplicates. Two independent reviewers [YB, AM] screened the records for eligibility in a two-step procedure: first based on the title and abstract, secondly, based on the full text. Rayyan application (22) was used in both screening step and helped compare the reviewers’ results, discuss discrepancies, and get consensual agreement. Two other reviewers [MTNN, IGC] managed weekly discussion sessions and cross-validated final article selection.

### 2.3 Data extraction and management

Data were extracted as defined in the protocol by two independent reviewers [YB, MTNN] and cross-checked by a third reviewer [IGC]. A particular attention was paid to the PROM identification and description (e.g., authors and publication year, version, number of dimensions and items, score calculation, cut-offs, and reporting).

#### 2.3.1 Evaluation of content validity

The methodological quality in developing PROMs and ensuring content validity was assessed following the COSMIN manual (18). For this, additional literature search was conducted for each identified PROM and the PROM’s authors were contacted by email to ensure the search completeness.

According to the COSMIN manual (18), the evaluation of PROM development quality encompasses 35 criteria distributed across two domains: (1) the quality of PROM design, incorporating the concept elicitation study for item generation, and (2) the quality of the cognitive interview study to evaluate the relevance, comprehensiveness, and comprehensibility of PROM items. Each criterion received a rating on a four-point scale: ‘very good,’ ‘adequate,’ ‘doubtful,’ or ‘inadequate.’ An additional set of COSMIN criteria was employed to evaluate the methodological rigor of studies focusing on content validation. A comprehensive assessment, consisting of 31 standards, scrutinized studies that reported relevance, comprehensiveness, or comprehensibility as perceived by either patients or professionals corresponding to the PROM’s target group. Each standard underwent rating on a four-point scale: ‘very good,’ ‘adequate,’ ‘doubtful,’ or ‘inadequate.’

Total scores were computed for both segments of the PROM development study, namely the quality of PROM design and the quality of the pilot testing study, usually based on cognitive interviews. Additionally, total scores were determined for each aspect of the methodological quality in content validation studies, including relevance, comprehensiveness, and comprehensibility. The scoring of each of these components was based on the lowest grade assigned to any evaluation criteria within that specific component (following the ‘the worst score counts’ principle) (18).

#### 2.3.2 Evidence synthesis

Initially, the outcomes of the study on PROM development, the examination of content validity, and the evaluations provided by reviewers regarding PROM content were assessed based on the 10 established criteria for robust content validity [15]. Five of these criteria concern the PROM’s content relevance, one the comprehensiveness, and four the comprehensibility (18). Each criterion was scored as sufficient (+), insufficient (−), or indeterminate (?). The findings from the available studies were then qualitatively summarized and evaluated based on the established criteria, ensuring sound content validity (18). Finally, a modified Grading of Recommendations, Assessment, Development, and Evaluations (GRADE) approach was employed to gauge the quality of evidence (23). Factors considered included the quality of the studies, the consistency of results across different studies, and the indirectness of evidence. The reviewers [YB, AM, MTNN] assessed the quality of evidence based on these factors, assuming high-quality evidence. Then, reviewers with the supervision of a fourth reviewer [IGC], downgraded the quality of evidence of PROMs one or two levels for each factor, if they are risk of bias, inconsistency, or indirectness.

## 3 Results

**Figure 1:**
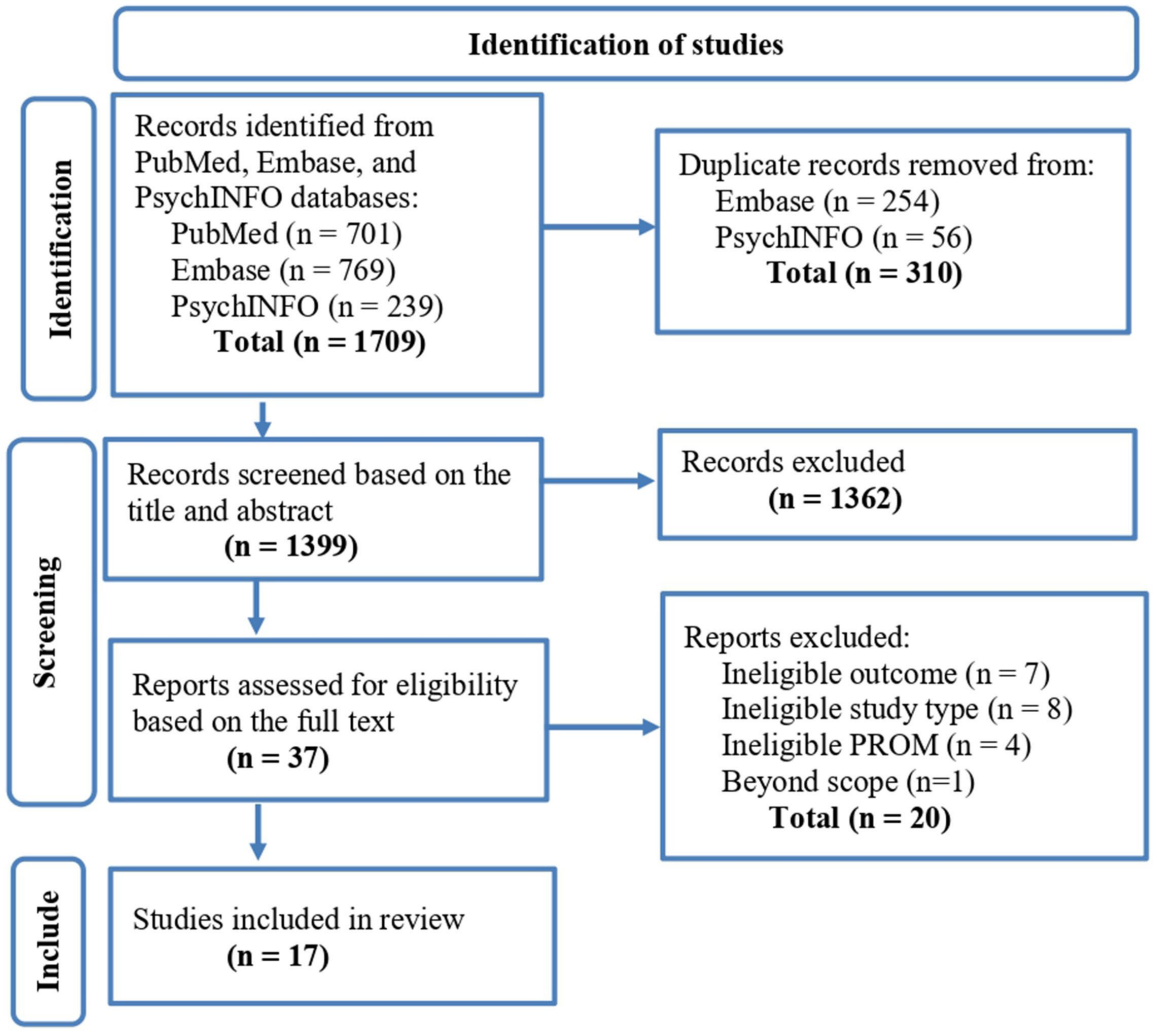
PRISMA floe diagram of study selection.

The literature search resulted in 1709 items. Following the elimination of duplicates, 1,399 records were considered in the first screening. Subsequently, during the second screening involving a comprehensive evaluation of 37 full-texts, 17 eligible articles were identified and reviewed (Figure 1).

### 3.1 Description of included studies

Table 1 summarizes the main characteristics of the included 17 studies. All studies have been conducted in high income countries, mostly in Australia. The sample size ranged between 60 and 1,445 participants of different occupations and specialties in animal care. Female participants were predominant (average sex ratio 80%) and the average age was around 40 years old. Most of the participants work in a clinical environment (64.7%), rather than research environment. The variation in the research objectives across studies but also in CF measures used presents a challenge in summarizing and comparing the study findings quantitatively (Table 1).

**Table 1:** Description of the included studies.

**Study reference (author, year and country)**	**Participants’ occupation** **(Sample size)**	**Sex ratio (Female %), Age in years, (Mea*n* ± SD)**	**Compassion fatigue measure (Name, reference)**	**Study aim**	**Main finding of the study**
Dow et al. (2019), Australia	Companion/ equine/ mixed animal in practice (*n* = 99), other animals (production/research) (*n* = 4)	63.1%, 24–75, (33.5 ± 13.4)	CFSS (Adams et al. 2006), 13 items	To determine the dealing with bereaved clients affected the psychological wellbeing of veterinarians	A significant proportion of veterinarians felt their own mental health was affected by dealing with clients grieving the loss of a companion animal.
Reif-Stice et al. (2023), United States	Currently practicing veterinary medicine (*n* = 223), Not currently practicing (*n* = 5), Retired (*n* = 2)	95.0%, NR	CFSS (Adams et al. 2006), 13 items	To assess the relationships between disclosure and responsiveness on anxiety, depression, and CF symptoms	There has a small, negative, direct relationship between disclosure and depression. And, the responsiveness had a significant, indirect effect on the relationship between disclosure and CF, anxiety, and depression.
Yeung et al. (2017), New Zealand	Wildlife carers (*n* = 17), Veterinary student (*n* = 6), Veterinary nurse (*n* = 2), Other (*n* = 2), Veterinarian (*n* = 1), Zookeeper (*n* = 1), Technician (*n* = 1)	77.0%, 20–70+, (NR)	ProQOL-5 (Stamm, 2009), 30 items	To assess CS and CF	Mea*n* ± SD in CS = 40.23 ± 6.56, Mea*n* ± SD in BO = 23.53 ± 5.78, Mea*n* ± SD in STS = 24.33 ± 5.44
Pizzolon et al. (2019), United States	Veterinary technician (*n* = 54), Client service representative (*n* = 52), Associate veterinarian (*n* = 37), Veterinary assistant (*n* = 20), Other (*n* = 16), Practice manager (*n* = 15), Registered veterinary technician (*n* = 15), Practice owner or partner (*n* = 12), Kennel assistant (*n* = 11)	84.5%, 18–73, (36.1 ± 11.9)	ProQOL-5 (Stamm, 2009), 30 items	To examine veterinary team effectiveness and personal empathy for associations with ProQOL and JS	Toxic team environment was positively associated with BO and negatively associated with JS. Empathetic concern and personal distress were positively associated with STS. Empathetic concern was moderated by team engagement for compassion satisfaction.
Andrukonis et al. (2020), United States	Kennel Attendant/ Animal Care Tech (*n* = 29), Vet/Vet Tech/Medical (*n* = 22), Other (*n* = 22), Animal Shelter Supervisor/Manager (*n* = 21), Animal Control Officer (*n* = 18), Shelter Director (*n* = 16), Customer Service Representative (*n* = 13), Behavior/ Training (*n* = 9), Assistant Director (*n* = 4), Volunteer/Rescue/ Community Coordinator (*n* = 3)	NR	ProQOL-5 (Stamm, 2009), 30 items	To assess the relationship between a shelter’s Live Release Rate (LRR) and the involvement in euthanasia-related decision making on employees’ mental health	CS, STS, moral injury, and BO were positively correlated with LRR. Employees who euthanize have higher moral injury scores compared with those who do not.
Perret et al. (2020a), Canada	Small animals only (*n* = 799), Mixed clinical practice (*n* = 141), Large animals only (*n* = 122), Equine only (*n* = 37)	78.4%, 25–73 (44.2 ± NR)	ProQOL-5 (Stamm, 2009), 30 items	To investigate the association of resilience with mental health outcomes	The resilience had negative associations with perceived stress, anxiety, depression, burnout, and secondary traumatic stress.
Perret et al. (2020b), Canada	Owner (*n* = 36), Associate or Locum (*n* = 24)	65%, 29–64, (NS)	ProQOL-5 (Stamm, 2009), 30 items	To investigate the association between veterinarian mental health and veterinary client satisfaction	Higher client satisfaction was associated with poor veterinarian mental health states, while lower client satisfaction was associated with mental health scores suggesting wellness.
Monaghan et al. (2020), Australia	Paid animal care work (*n* = 256), Voluntary animal care work (*n* = 180), both voluntary and paid animal care work (*n* = 123)	94.9%, NR	ProQOL-5 (Stamm, 2009), 30 items	To assess CF risk levels and the extent to which job demands predict CF	No significant differences in CF risk between paid and volunteer animal carers. Job demands predicted 18% in STS and 17% in B
Goñi-Balentziaga et al. (2021), Spain	Investigators (*n* = 103), PhD students (*n* = 99), Animal caretaker/ technicians (*n* = 97), Principal investigators (*n* = 69), Research technicians (*n* = 68), Welfare officers/ veterinarians (*n* = 62)	67.3%, 21–69, (36 ± 10.4)	ProQOL-5 (Stamm, 2009), 30 items	To investigate the work-related quality of life	Animal-facility personnel showed higher total ProQoL and compassion-satisfaction scores than researchers while PhD students showed the lowest ProQoL scores.
Schlanser et al. (2021), United States	Veterinary laboratory animal medicine (*n* = 35), Animal care specialist (*n* = 28), Field veterinary service officer (*n* = 2),	68.0%, NR	ProQOL-5 (Stamm, 2009), 30 items	To describe the prevalence of CF	Mea*n* ± SD in BO = 20.83 ± 6.84, Mea*n* ± SD in STS = 18.95 ± 5.80
Signal et al. (2022), Australia	Animal rescuers (*n* = 342)	92%, 18–77, (41.2 ± 14.1)	ProQOL-5 (Stamm, 2009), 30 items	To assess CF by using the ProQOL	In an Australian study of animal rescuers, a significant prevalence of compassion fatigue symptoms was observed. Higher levels of compassion fatigue were associated with symptoms of depression and increased anxiety, highlighting potential negative impacts on mental wellbeing.
Rohlf et al. (2022), Australia	Veterinary nurses (*n* = 93), Veterinarians (*n* = 43)	94.9%, NS	ProQOL-5 (Stamm, 2009), 30 items	To explore personal and organizational factors predicting CS and CF, and the linkage with their current role and profession	Personal factors accounted 31.1% in CS, 45.3% in BO, and 33.8% in STS. Organizational factors accounted for 33.3% in CS, 47.9% in BO, and 32.7% in STS. ProQOL accounted for 28.9% to leave one’s current role and 16.0% to leave the profession.
Musetti et al. (2020), Italy	Companion animal practice (*n* = 1,227), Production animals (*n* = 69), Other (*n* = 62), Veterinary local units (*n* = 49), University staff (*n* = 23), Pharmaceuticals (*n* = 15)	70.0%, 24–74, (43.27 ± 11.1)	Adapted ProQOL (Palestini et al. 2009, Stamm, 2009), 22 items	To examine the contextual factors related to the work of veterinarians, and the individual factors related to the security of approach to life and relationship contributed to the quality of life	Female gender, higher levels of ordinary workload, on-call hours per week, exposure to animal suffering, together with fearful and preoccupied attachment styles were significantly associated with lower levels of veterinarians’ quality of life.
Polachek et al. (2018), Canada	Animal health care providers (*n* = 572)	82%, NR	Shortened version of ProQOL (Stamm, 2009),9 items	To explore the paradox of compassionate work by examining what interactions contribute to CS and CF	Human client barriers to animal care and witnessing client grief relate to CF. Forming relationships with animal patients relates to both CS and CF
Hess-Holden et al. (2019), United States	Fourth-year veterinary students (*n* = 281)	89.7%, NR	ProQOL-5: Veterinary Medicine Version (Stamm, 2009), 30 items	To investigate the relationship between compassion experiences and communication styles	CF was associated with the communication styles of emotionality, impression manipulativeness, and verbal aggressiveness.
Randall et al. (2021), Canada	General animal professionals (*n* = 154), Contract Research Organizaiton Laboratory animal professionals (*n* = 268)	73%, 18–65+, (NR)	The CF Questionnaire (Randall et al. 2021), 30 items	To investigate compassion fatigue (CF), explore the impact of personal and work-related factors on CF, evaluate coping mechanisms employed to address CF, and identify the beneficial components of a CF support program	Compassion fatigue (CF) was found to be prevalent at 69%. Factors contributing to CF included understaffing, close relationships with experimental animals, insufficient resources for coping, poor relationships with superiors, and a lack of training in managing CF. Beneficial coping mechanisms identified were talking to a trusted individual, taking breaks from work, implementing self-care strategies, increasing engagement in physical activities, and owning or caring for companion animals.
O’Malley et al. (2022), United States	Veterinarians (*n* = 120), Research personnel (*n* = 99), Animal care personnel (*n* = 97), Other (*n* = 32), Study director (*n* = 31), Veterinary support (*n* = 23), Pathologist (*n* = 4), Necropsy (*n* = 3)	69%, 18–65, (NR)	The CF Questionnaire (Randall et al. 2021), 30 items	To assess CF in and across employment categories	Prevalence of CF is 52% from the EU, 56% from China and 32% from Japan. No major differences were found based on employer type.

NR, not reported, STS, secondary traumatic stress; BO, burnout; CS, compassion satisfaction; CF, compassion fatigue; ProQOL, professional quality of life; JS, job satisfaction.

### 3.2 Description of identified CF measures

In total, six different PROMs or their versions for assessing CF were identified across the reviewed studies (Table 2). These PROMs are the Professional Quality of Life-Version 5 (ProQOL-5) (*n* = 12), the Compassion Fatigue Short Scale (CFSS)(*n* = 2), the compassion fatigue questionnaire (*n* = 2) and ProQOL-5 Veterinary Medicine Version (ProQOL-5-VMV) (*n* = 1). Conversely to its title, the CF questionnaire contains only one item dedicated to CF assessment. The CF is there defined as follows: “Compassion fatigue is a profound emotional and physical exhaustion that people in the caring professions can develop when they are unable to refuel and regenerate due to the nature of their work. CF is a normal occurrence and is commonly seen across many professions including, but not limited to, nurses, hospice workers, veterinarians and veterinary technicians, social workers, and laboratory animal caregivers.” and the item reads “Have you ever experienced compassion fatigue?,” with three response options: Yes, No, and Unsure. The other 29 items address the participant’s demographics, the influence of personality on CF, the nature of work, and coping mechanisms. Therefore, the CF questionnaire cannot be considered a PROM and was excluded from the second review of PROMs’ content validity.

**Table 2:** Summary of the identified measures of compassion fatigue in animal care professionals studies.

CF Measure, Reference	Language	Number of dimensions and items	Recall period	Response option	Total score range
CFSS (Compassion fatigue short scale), Adams et al. (2006)	English	2 dimensions STS (5 item), BO (8 item)	Not defined	10-point likert scale (Never-very often)	13–130
ProQOL-5 (Professional quality of life-version 5), Stamm (2009)	English	3 dimensions STS (10 items), BO (10 items), CS (10 items)	30 Days	5-point likert scale (Never-very often)	30–150
Adapted ProQOL, Palestini et al. (2009)	Italy	3 dimensions CF (7 items), BO (7 items), CS (8items)	30 Days	5-point likert scale (Never-very often)	22–110
Shortened ProQOL-5, Polachek et al. (2017)	English	2 dimensions CS (3 items), CF (6 items)	30 Days	5-point likert scale (Never-very often)	Not defined
ProQOL: Veterinary Medicine Version, Hess-Holden et al. (2019)	English	3 dimensions STS (10 items), BO (10 items), CS (10 items)	30 Days	5-point likert scale (Never-very often)	30–150
The CF questionnaire, Randall et al. (2021)	English	One item of	Not defined	The author provided the definition of CF and asked “Have you ever experienced CF?” with the response option of “Yes, No, Unsure.”	Not defined

STS, secondary traumatic stress; BO, burnout; CS, compassion satisfaction; CF, compassion fatigue, ProQOL, professional quality of life.

A ProQOL-5 modified version (24) was neither considered as it was not developed specifically or independently on ProQOL-5 but rather shortened ProQOL-5 for administration-facilitating purposes and without proper development and validation studies. A translated in Italian ProQOL-5 version (25) was also omitted, since within the COSMIN framework, content validation takes precedence over cross-cultural validation of a PROM (26). It is assumed that the content validity of a validly translated version would be like that of original version. Both CFSS and ProQOL originated from the Compassion Fatigue Self-Test (CFST) (2). When a PROM is originated from an earlier version of the scale, COSMIN guidelines suggest evaluating the current PROM with its ancestor. In cases when PROM is adapted to be used in another target population than the original target population, it is crucial to reassess the content of both the adapted and the original version (18, 26) in case it was not done before. This procedure was followed to ensure whether adapted version represents the CF construct for the given target group. By assessing the original PROM (CFST) along with the adapted version (ProQOL), we were able to better capture the content of originally selected items and assess their relevance and comprehensiveness with respect to the theoretical foundations of CF construct features. Therefore, the CFST was included in the content validity assessment.

### 3.3 Construct definition and description of the reviewed PROMs

#### 3.3.1 Compassion fatigue self-test (CFST)

The conceptualization of CF that underlines the CFST corresponds to “the natural consequent behaviors and emotions resulting from knowing about a traumatizing event experienced or suffered by a person” (2). Figley pioneered the development of a tool to assess CF, and the CFST for practitioners was introduced in his book “Compassion Fatigue: Coping with Secondary Traumatic Stress Disorder in Those Who Treat the Traumatized” in 1995. The original CFST scores were based on data from 142 psychotherapy practitioners who participated in workshops on CF between 1992 and 1993. However, information about the characteristics of this sample and the study design is not available.

CFST was developed to introduce the concept of CF in workers with traumatized clients, establish a theoretical basis for assessing and treating compassion stress and fatigue, explain the difference between CF and Post-traumatic stress disorder (PTSD), burnout, and other related phenomena, identify innovative methods for treating CF in therapists, and suggest methods for preventing CF. The CFST evaluates CF and job burnout (BO) using 40 items across two subscales: CF (23 items) and BO (17 items). The target population is trauma workers engaged with traumatized individuals regularly. Respondents rated the frequency of traits or situations concerning themselves on a scale from 1 (rarely/never) to 5 (very often). Scores on the CF subscale below 26 indicate extremely low risk, 27 to 30 suggest low risk, 31 to 35 indicate moderate risk, 36 to 40 indicate high risk, and scores of 41 or above indicate an extremely high risk of CF. On the burnout subscale, scores between 17 and 36 or less suggest extremely low risk, 37 to 50 suggest moderate risk, 51 to 75 suggest high risk, and 76 to 85 indicate extremely high risk of burnout (2). However, information about the development, amendment, and validation of this measure is not provided in the book chapters or other publications.

#### 3.3.2 Compassion fatigue short scale (CFSS)

The CFSS is a shortened and revised version of the CFST (27). It was designed by Adams in 2006 for formal caregivers, specifically targeting first responders in clinical settings (28). CFSS is built on the concept that CF represents a caregiver’s diminished ability or interest in being empathic or “bearing the suffering of clients” (28). Adams stated that research on CF has had several problems. First, there has been a lack of conceptual clarity about what constitutes CF and how it differs from other adverse work outcomes, such as job burnout. Indeed, no study has fully incorporated all aspects of Figley’s description of CF. To address these gaps, Adams focused on social workers in clinical practice in a region affected by a major traumatic event- the September 11, 2001, terrorist attacks in New York City. CFSS was connected to the work environment of social workers because of high caseloads and inadequate resources (28). CFSS construct includes two dimensions: secondary traumatic stress (STS) measured with a 5-items subscale and BO (8 items). CFSS total scores can vary from a minimum of 13, indicating no symptoms of CF, to a maximum score of 130 for individuals experiencing frequent symptoms of CF.

#### 3.3.3 Professional quality of life version 5 (ProQOL-5)

The ProQOL was developed as an evolution and expansion upon the CFST. ProQOL and its five subsequent versions consist of three subscales: BO, STS, and Compassion satisfaction. The transition from using the term CF to STS in ProQOL-5 reflects an evolution in terminology. However, specific details regarding the timing and individuals involved in this change are not explicitly known. This modification was likely influenced by ongoing research findings and emerging insights within the field. The ProQOL was designed to provide a more comprehensive assessment of the overall quality of life of individuals working in helping professions, incorporating not only the negative aspects like STS and BO but also the positive aspects such as compassion satisfaction. The target population is the people who work in helping professions that may respond to individual, community, national, and even international crises (3).

ProQOL-5 is the latest version of ProQOL (29). The measure demonstrates a high popularity, as indicated by its extensive use and a substantial online presence. However, the author did not provide details about its content validity.

#### 3.3.4 Professional quality of life version 5 veterinary medicine version (ProQOL-5-VMV)

Hess-Holden conducted a modification of the ProQOL-5, resulting in the ProQOL-5-VMV tailored for veterinary professionals (30). This adaptation involved adjusting wording and potentially the content to align with the experiences of these professionals. The study involved 281 fourth-year veterinary medicine students in the United States, focusing on the relationship between compassion experiences and communication styles. This study is a descriptive study, and it does not encompass a content validation study or any other types of validation beside the Cronbach alpha estimation.

### 3.4 Quality of the PROMs development studies

PROM development studies were found for only two PROMs (CFST and ProQOL-5). As already mentioned, the CFSS was derivative of the CFST and lacks an independent development study. The ProQOL-5-VMV was introduced in a study as a CF measure, only describing that it has an adjusted wording of ProQOL-5 to align with the experiences of the veterinarians. Therefore, the assessment of these PROMs’ development quality was not possible, and we rated it as doubtful.

The design requirements for the CFST and ProQOL-5 were judged inadequate due to insufficient details concerning the constructs to be measured, the context of use, and deficiencies in concept elicitation information. There were uncertainties about the representativeness of participants included in the CFST and ProQOL-5 development, as it remained unclear whether they truly represented the target population. In CFST, the development process involved 142 psychotherapy practitioners who participated in workshops on CF between 1992 and 1993, but description of this sample is not provided, and it is does not encompass all representatives of target population. In ProQOL-5, the target population is the people who work in helping professions that may respond to individual, community, national, and even international crises but ProQOL-5 used the term of “helpers” which is ambiguous and could diminish the professional life in this capacity. Furthermore, the development processes of the CFST and ProQOL-5 lacked clear descriptions of the methods used, particularly regarding the cognitive interviews with participants. Moreover, no details were provided regarding whether and how the comprehensibility and comprehensiveness have been assessed. Although we contacted both PROMs’ first authors, no additional details were obtained besides what we found in the literature. Thus, the PROM development studies for CFST and ProQOL-5 were also considered of doubtful quality.

### 3.5 Quality of the patient reported outcome measure content validity

The evaluation of the content validity involves assessing three key criteria: relevance, comprehensiveness, and comprehensibility. Relevance ensures that PROM items are tailored to both the target population and the specific context of use. Comprehensiveness examines the overall completeness of the PROM, while comprehensibility focuses on how well the target population understands the PROM.

In our study, the CFSS is the single PROM for which a content validation study was available. This study was conducted in 2019 among 132 Turkish healthcare workers. This study included professionals from diverse fields such as nursing, medicine, psychology, and social work, covering various clinics such as neurology, infection, surgery, psychiatry, and emergency services in two hospitals located in Istanbul (31). The result suggested that the CFSS is a reliable and valid scale in the context of Turkish healthcare professionals (31). However, the authors did not provide information on the use of an interview/topic guide, involvement of trained moderators/interviewers, or the employment of two independent researchers for data analysis, as required in COSMIN manual (18). Consequently, the quality of this content validation study was rated as insufficient.

### 3.6 Grading of the overall quality of evidence

In evaluating the four PROMs included in this review, no high-quality evidence was identified, as indicated in Table 3, bottom line. All PROMs received low-quality evidence for relevance, comprehensiveness, and comprehensibility. For relevance, all PROMs received low-quality evidence rating because the CFST and CFSS were assessed as having insufficient content validity, while ProQOL-5 and its veterinary medicine version were rated to have inconsistent content validity quality. The evidence quality for comprehensiveness received a low rating for all PROMs, with CFST having insufficient content validity, and other PROMs having indeterminate content validity. Similarly, the evidence quality for comprehensibility was low for all PROMs, with assessments indicating indeterminate content validity (Table 3).

**Table 3:** Results of content validity assessment in compassion fatigue measures included in the systematic review.

CF **measure**	**CFST**	**CFSS**	**ProQOL-5**	**ProQOL-Veterinary Medicine Version**
First author, year	Figley (1995)	Adams et al. (2006)	Stamm (2009)	Hess-Holden et al. (2023)
***Relevance***	***Insufficient***	***Inconsistent***	***Inconsistent***	***Inconsistent***
Relevant for the construct of interest	Inconsistent	Insufficient	Inconsistent	Inconsistent
Relevant for the target population of interest	Insufficient	Inconsistent	Inconsistent	Sufficient
Relevant for the context of use of interest	Inconsistent	Inconsistent	Inconsistent	Inconsistent
Appropriate response options	Sufficient	Sufficient	Sufficient	Sufficient
Appropriate recall period	Indeterminate	Sufficient	Sufficient	Indeterminate
***Comprehensiveness***	***Insufficient***	***Indeterminate***	***Indeterminate***	***Indeterminate***
Include all key concepts	Insufficient	Indeterminate	Indeterminate	Indeterminate
***Comprehensibility***	***Inconsistent***	***Inconsistent***	***Inconsistent***	***Inconsistent***
Are the PROM items appropriately worded?	Sufficient	Sufficient	Sufficient	Sufficient
Do the response options match the question?	Indeterminate	Indeterminate	Inconsistent	Inconsistent
**Overall content validity rating**	***Insufficient***	***Insufficient***	***Inconsistent***	***Inconsistent***
**Quality of evidence**	***Low***	***Low***	***Low***	***Low***

## 4 Discussion

### 4.1 Main findings

In this study, and for the first time, we systematically assessed the CF measures used in animal health care professionals and examined the content validity according to the COSMIN guidelines in four identified PROMs. According to study findings, the content validity of these PROMs is not sufficient to recommend any of them as a valid PROM for measuring CF in research or animal care practice. The overall quality of the evidence is low, meaning that further studies are necessary to validate and compare these PROMs, enabling the identification of the most valid and recommendable one.

### 4.2 Methodological limitations in included studies

The systematic review revealed notable shortcomings in evaluating content validity for measuring CF. Methodological deficiencies were observed in four PROMs to measure CF. These limitations included a lack of clarity regarding the constructs being measured, insufficient specificity regarding the context, deficiencies in the process of item elicitation, uncertainties regarding the representation of the target population, and inadequate descriptions of the cognitive interview process used to assess the comprehensibility and comprehensiveness of the PROMs. The utilization of PROMs like CFST (1995), CFSS (2006), ProQOL-5 (2010), and ProQOL-5 VRV (2018), occurred before the introduction of established methodological standards such as the COSMIN content validity criteria in 2018 (18). The reliance on older instruments likely contributed to suboptimal methodological practices and inconsistent reporting within the studies (32). Recommendations include involving animal health professionals in item elicitation and using cognitive interviews to improve the relevance, comprehensiveness and comprehensibility of the PROM content with respect to the CF measurement in the target population. High content validity can be accomplished by conducting interviews or focus groups with representatives of target population, ensuring the language used in the PROM aligns with theirs, and incorporating the content of their qualitative statements about CF (33). Additionally, adhering to established guidelines such as the Consolidated Criteria for Reporting Qualitative Studies (COREQ) is crucial for reporting future content validation studies (34), where the qualitative research methods better suit the validation purpose, as stated in the PROM development guidelines (35–37). Addressing these limitations would lead to a more comprehensive and rigorous evaluation of CF among animal health professionals.

### 4.3 Challenges in CF research and PROM development

#### 4.3.1 Limitations inherent to the CF studies in animal health care professionals

Despite an extensive literature search and multiple linguistic options, the reviewed studies predominantly originate from developed English-speaking countries, notably Australia. Therefore, the generalizability of their findings to a broader range of animal health care professionals globally is questionable. The substantial overrepresentation of women, comprising 80% of study participants, reflects the feminization trend observed in the veterinary profession, as supported by data indicating that 87.3% of current veterinary school applicants (38) and 88% of veterinary technicians (39) identify as female. While it’s possible that females are more affected by compassion fatigue and therefore participated more in studies, it’s important to acknowledge the need for a comprehensive understanding of CF that considers the experiences and coping mechanisms of all individuals, including male counterparts. Moreover, the average age of participants being approximately 40 years may skew the results toward a specific age group, failing to capture the diversity of experiences and coping strategies among younger and older animal health care professionals. These features point out that CF investigation in animal health care professionals is still at its early stage and could improve.

Furthermore, we noticed an inconsistent use of CF measurement methods and statistical approaches, leading to a heterogenous result reporting, which precluded the use of meta-analysis to quantitively summarizing the prevalence of CF in animal health care professionals. Indeed, only two studies (40, 41) out of 17 included reported CF prevalence based on CF questionnaire, whereas other studies used CF PROMs without applying predefined cutoff values to estimate CF prevalence. Such an inconsistency in result reporting might be partly due to varying research objectives across included studies. However, it could be also due to lacunary or missing guidance regarding score calculation and result reporting in existent PROMs. For example, the CFSS validation study (31) provides no guidance for calculating the “Obtainable score” and it seems unclear whether the score should be reported per subscale or overall. Notwithstanding, despite Stamm’s guidance on the need of t-transformation of the ProQOL-5 scores and the cutoff values provided to estimate high CF level (3), the authors have not consistently adhered to these instructions when using the ProQOL-5 (24, 30, 42–51), resulting in compromised comparability of CF results.

Marca et al. (52) highlighted the critical importance of validity in validating mental health questionnaires and rating scales and the lack of standardized validation processes, leading to uncertainties and limitations in terms of validity. Key issues reported by Marca et al. (52) include the importance of clarity in terminology, standardization of statistical assessment methods, and the establishment of universally agreed-upon guidelines. The ambiguity in crucial terms like reliability, validity, and content and construct validity poses challenges in the PROM validation and thus in assessment of mental health problems (52). Addressing these core issues is important for improving the assessment and prevention of psychological disorders.

#### 4.3.2 Challenges in standardization and development of PROMs

Developing PROMs tailored for CF poses distinct challenges, particularly in capturing the nuanced emotional experiences of animal health workers within this domain. While numerous PROM development guidelines exist, as evidenced by studies from Morgado et al. (35), Boateng et al. (36), and Kyriazos et al. (37), adherence among researchers remains inconsistent. The study by Morgado et al. sheds light on common limitations in PROM scale development, stressing the importance of establishing a solid theoretical foundation, conducting thorough construct validation, ensuring adequate sample sizes, and employing comprehensive item selection procedures (35). Similarly, Boateng et al. (36) emphasizes transparency in reporting, meticulous testing for convergent and divergent validity, careful selection of response scales and item wording, and assessment of measurement invariance. Kyriazos et al. (37) further underscores the necessity of creating a robust theoretical framework, validating constructs, determining appropriate sample sizes, and ensuring transparent reporting.

Various guidelines have been established to provide recommendations for ensuring content validity in PROM development, including conducting literature reviews, utilizing concept elicitation or focus groups, analysing data, generating items, and conducting cognitive interviews (33, 35–37, 53–55). However, the extent to which these guidelines are followed in practice varies among researchers. Given the variability in adherence to PROM development guidelines, it is imperative to advocate for their consistent utilization in future research endeavors (26, 32, 56). Following these guidelines developing and testing PROMs tailored for CF and testing will enhance their content and overall validity. Subsequently, the content validity of these PROMs can be easier assessed using established frameworks such as the COSMIN guidelines (18) and help compare available PROMs and identify the most valid one.

### 4.4 Limitation in compassion fatigue definition

CF demonstrates significant variability in its definition across different authors and scholars. For instance, Joinson characterizes CF as a unique form of burnout (1). Conversely, Figley suggests that CF is interchangeable with secondary traumatic stress (STS), indicating a close relationship with exposure to trauma through empathic engagement (2). Adams contributes to the discourse by highlighting the lack of clarity surrounding CF, further complicating its conceptualization (28). VanMoi et al. portrays CF as an ongoing and snowballing process, emphasizing continuous exposure to distressing situations that lead to moral distress and emotional depletion (57). Finally, Rauvola et al. (58) defines CF as an acute onset of emotional exhaustion and detachment, suggesting it is part of the broader category of empathy-based stress outcomes. Amidst these varying perspectives, Eng’s study underscored the need for clarification and exploration of the relationships between CF, burnout, and STS, culminating in the development of a new measurement tool, the Compassion Fatigue Inventory (CFI) (59). This diversity in CF definitions contrasts with a relative communality in the CF construct and measures despite a growing criticism among CF researchers and some clearly distinctive features mentioned above. This highlights the need of a better understanding and conceptualization of CF in general and in some specific professions particularly, prior to its harmonized definition and PROM development. This situation is not unique to CF. It’s worth reminding that burnout definition has remained for a long time vague and inconsistent and been harmonized only recently (60, 61). This harmonization along with a systematic assessment of burnout measures (62, 63) enabled researchers assessing and meta-analyzing burnout prevalence in different countries and occupational groups (5, 64, 65), which was impossible before despite a 60-year research on the topic. The CF concept appeared in the 90ths, and the empiric evidence and scientific literature are less abundant. Thus, the paramount question to answer is whether this form of stress is different from the other forms of occupational stress (e.g., burnout, post- and secondary traumatic stress syndrome, moral distress) and whether it can be really measured independently, using a specific PROM. Further research following Rauvola’s et al. (58) recommendations could hopefully answer these questions.

### 4.5 Challenges in assessing content validity related to the COSMIN method application

The concept of content validity is a topic of ongoing discussion in the scientific community, with varying perspectives on its definition and evaluation (18, 32, 66). In our study on CF among animal health workers, evaluating content validity posed unique challenges that were intricately connected with existing literature. A significant challenge we encountered was distinguishing between cognitive interviews and content validation studies, which was exemplified by inconsistencies in reporting practices across studies. For example, CFST development and test involved 142 psychotherapy practitioners, but the report lacked description of the sample’s representativeness (2), while CFSS’ pilot test lacked crucial details as required by COSMIN (31). While these studies mentioned interviews with participants, details regarding the interview process, such as the number of interviewers and specific prompts used, were often lacking, hindering our ability to ascertain their contribution to content validity (31, 56, 67, 68). Additionally, aligning PROM items with intended constructs across different studies proved challenging, necessitating meticulous scrutiny and interpretation of available data (31, 56, 67–69). The evolution of CF measurement added another layer of complexity, with early instruments like CFST assuming that CF is interchangeable with STS (2), while later instruments like CFSS separated these concepts (28) and the ProQOL-5 introduced compassion satisfaction (CS) as another distinct construct (29). This inconsistency in subscale labeling and definitions along with potential overlap between concepts within existing tools made it challenging to delineate CF from related experiences and requires careful consideration when dealing with multidimensional PROMs (32, 56, 68). Criticisms have been raised regarding the perceived strictness of the worst-score-counts principle, potentially leading to the issuance of ‘doubtful’ ratings withing the COSMIN framework (32). Despite the COSMIN methodology being widely recognized as the benchmark for PROM quality evaluation (31, 56, 67–69), overcoming these challenges is crucial to ensure its effective implementation.

### 4.6 Study strengths

The study boasts several notable strengths. First, it adhered to a robust research protocol, which allowed us to maintain research rigor and transparency throughout the entire review processes, starting with the pre-registration of the protocol in PROSPERO and its strict application during all review steps. Secondly, the study involved an experienced librarian who performed a comprehensive literature search spanning 50 years across three databases (PubMed, PsycINFO, and EMBASE), thereby ensuring the inclusion of a wide range of relevant studies. Thirdly, studies reporting at least one psychometric property of the employed CF measure, not just the content validity, were considered eligible. Thereby we could minimize the risk of missing relevant evidence related to CF measurement. Fourthly, the screening and data extraction process were executed meticulously by three independent reviewers, with results cross-checked by a fourth reviewer, ensuring the accuracy and reliability of the findings. Finally, the COSMIN methodology used in the study has been proven effective in evaluating the content validity of PROMs in various medical specialties (4, 32, 56, 62, 67, 70).

### 4.7 Study limitations

Our systematic review, like any review relying on secondary data, has inherent limitations. These limitations stem from two key areas: dependence on existing research and the complexities of assessing CF measurement tools. Firstly, the quality and completeness of the included studies can influence our findings. Potential biases within these studies, such as publication bias or methodological shortcomings, are beyond our control. We mitigated this risk by employing a rigorous search strategy and established criteria for assessing methodological quality. Secondly, the studies themselves exhibited limitations in evaluating CF measurement tools, particularly regarding content validity. Psychometrics, the field concerned with measurement properties, is more complex than clinometrics. Therefore, inadequate reporting practices in the primary studies made it difficult to accurately assess content validity using the COSMIN framework. Furthermore, the lack of clarity in target constructs and inconsistent reporting practices within the primary studies themselves limited our ability to definitively assess the content validity of the included CF measurement tools. Finally, the concept of CF itself presents a challenge. The ongoing debate around its definition and construct adds another layer of complexity. This highlights the need for future research to establish standardized methodologies and clearer definitions, ultimately enhancing the reliability of findings in CF research.

## 5 Conclusion

In conclusion, the study findings highlight both the insufficiency of robust evidence supporting the content validity of current PROMs used for measuring CF in animal health care professionals and other settings, alongside the recognition of the scarcity and underutilization of tailored PROMs for this target population. This insufficiency is primarily rooted in concerns regarding the quality of development studies, the adequacy of content validity in existing PROMs, and the unclear definition of the CF concept and construct. Of particular concern is the continued utilization of these PROMs without periodic reassessment.

The Culture of Care is crucial nowadays, representing a commitment to animal welfare, scientific quality, staff well-being, and transparency (71). Additionally, experiencing CF and its negative consequences can have an impact on the well-being of animals, particularly in the context of utilizing the 3R principle (replacement, reduction, refinement) in research involving animals. Caretakers play a crucial role in promoting these principles, and research has shown a correlation between a decreased quality of life among laboratory animal staff and their lack of control over euthanasia procedures (16).

The future research efforts should focus on a better understanding and harmonization of the CF concept definition and construct underpinning with a stronger theoretical framework. Additionally, adherence to standardized guidelines for PROM development and validation is crucial to ensure sufficient validity in existing and newly developed PROMs, thereby improving their utility in clinical practice and research.

## Data availability statement

The original contributions presented in the study are included in the article/supplementary material, further inquiries can be directed to the corresponding author.

## Author contributions

MTNN: Formal analysis, Project administration, Resources, Validation, Visualization, Writing – original draft, Writing – review & editing, Funding acquisition, Investigation, Methodology. YB: Conceptualization, Data curation, Investigation, Methodology, Software, Validation, Writing – review & editing, Funding acquisition, Resources, Visualization. AM: Conceptualization, Data curation, Formal analysis, Investigation, Methodology, Software, Validation, Visualization, Funding acquisition, Resources, Writing – review & editing. SH: Conceptualization, Formal analysis, Funding acquisition, Investigation, Methodology, Project administration, Resources, Supervision, Validation, Visualization, Writing – review & editing. IGC: Conceptualization, Data curation, Formal analysis, Funding acquisition, Investigation, Methodology, Project administration, Resources, Supervision, Validation, Visualization, Writing – original draft, Writing – review & editing.
